# Electrical Stimulation of Afferent Pathways for the Suppression of Pathological Tremor

**DOI:** 10.3389/fnins.2017.00178

**Published:** 2017-04-04

**Authors:** Jakob L. Dideriksen, Christopher M. Laine, Strahinja Dosen, Silvia Muceli, Eduardo Rocon, José L. Pons, Julian Benito-Leon, Dario Farina

**Affiliations:** ^1^SMI, Department of Health Science and Technology, Aalborg UniversityAalborg, Denmark; ^2^Brain-Body Dynamics Lab, University of Southern CaliforniaLos Angeles, CA, USA; ^3^Institute of Neurorehabilitation Systems, University Medical Center GöttingenGöttingen, Germany; ^4^Clinic for Trauma Surgery, Orthopedics and Plastic Surgery, University Medical Center GöttingenGöttingen, Germany; ^5^Center for Automation and Robotics, Consejo Superior de Investigaciones CientíficasArganda del Rey, Spain; ^6^Neural Rehabilitation Group, Instituto Cajal, Consejo Superior de Investigaciones CientíficasMadrid, Spain; ^7^Department of Neurology, University Hospital 12 de OctubreMadrid, Spain; ^8^Department of Bioengineering, Imperial College LondonLondon, UK

**Keywords:** afferent pathways, electrical stimulation, pathological tremor, neuromodulation, tremor suppression

## Abstract

Pathological tremors are involuntary oscillatory movements which cannot be fully attenuated using conventional treatments. For this reason, several studies have investigated the use of neuromuscular electrical stimulation for tremor suppression. In a recent study, however, we found that electrical stimulation below the motor threshold also suppressed tremor, indicating involvement of afferent pathways. In this study, we further explored this possibility by systematically investigating how tremor suppression by afferent stimulation depends on the stimulation settings. In this way, we aimed at identifying the optimal stimulation strategy, as well as to elucidate the underlying physiological mechanisms of tremor suppression. Stimulation strategies varying the stimulation intensity and pulse timing were tested in nine tremor patients using either intramuscular or surface stimulation. Significant tremor suppression was observed in six patients (tremor suppression > 75% was observed in three patients) and the average optimal suppression level observed across all subjects was 52%. The efficiency for each stimulation setting, however, varied substantially across patients and it was not possible to identify a single set of stimulation parameters that yielded positive results in all patients. For example, tremor suppression was achieved both with stimulation delivered in an out-of-phase pattern with respect to the tremor, and with random timing of the stimulation. Overall, these results indicate that low-current stimulation of afferent fibers is a promising approach for tremor suppression, but that further research is required to identify how the effect can be maximized in the individual patient.

## Introduction

Pathological tremors (henceforth referred to as tremor) are involuntary, rhythmical movements of a body part and are symptomatic of several neurological disorders including Parkinson's Disease (PD) and Essential Tremor (ET) (Elble, 2009). Tremor, that arises due to burst-like muscle activity patterns (Deuschl et al., 1987), is among the most prevalent movement disorders and can partly or completely impair the execution of natural motor tasks (Wenning et al., 2005). Standard treatment includes medication (Lyons and Pahwa, 2008), neurosurgery (Kondziolka et al., 2008), or deep brain stimulation (Kalia et al., 2013). Such treatments, however, can be invasive, expensive, and may not produce effective, long-lasting tremor suppression tremor for all patients. As a potential alternative, studies have suggested the use of external devices to suppress tremor by mechanical loading (Pledgie et al., 2000; Rocon et al., 2007) or electrical stimulation (Javidan et al., 1992; Prochazka et al., 1992; Gillard et al., 1999; Popović Maneski et al., 2011; Gallego et al., 2013; Bó et al., 2014; Dosen et al., 2015). Although the efficacy of both methodologies has been proven, electrical stimulation arguably allows for a more compact and comfortable implementation. Both of these factors are considered critical for minimizing user rejection of orthotic devices (Biddiss and Chau, 2007).

Table 1 summarizes the studies investigating the use of electrical stimulation for tremor suppression. Overall, two primary stimulation strategies have been applied. The most common strategy (*out-of-phase*) applies electrical stimulation to the muscles so that they generate forces opposite to those arising from the tremorogenic bursts of activity (Javidan et al., 1992; Prochazka et al., 1992; Gillard et al., 1999; Popović Maneski et al., 2011; Dosen et al., 2015). The other strategy (*cocontraction*) provides continuous stimulation simultaneously to antagonist muscles acting about the affected joint in order to increase the stiffness of the joint through co-activation, and thereby filter out the mechanical manifestation (joint oscillations) of the tremorogenic bursts (Grimaldi et al., 2011; Gallego et al., 2013; Bó et al., 2014). A common characteristic of both strategies is that they rely on classical neuromuscular electrical stimulation, in which pulse intensity is set high enough to activate efferent nerve fibers and elicit muscle contractions. This approach has several well-known drawbacks, such as a rapid development of fatigue in the stimulated muscles (Maffiuletti, 2010; Bickel et al., 2011), potential discomfort due to strong stimulation, and interference with voluntary movements, which may all impair the long-term effectiveness of the stimulation.

**Table 1:** **State of the art in suppression of tremors using electrical stimulation**.

**Reference**	**Patients**	**Joint**	**Strategy**	**Suppression**
Javidan et al., 1992; Prochazka et al., 1992	3 ET, 4 PD, 6 other	Wrist	Out-of-phase	53 ± 25%
Gillard et al., 1999	3 PD	Wrist/Finger	Out-of-phase	83 ± 2%
Popović Maneski et al., 2011	3 ET, 4 PD	Wrist	Out-of-phase	67 ± 13%
Grimaldi et al., 2011	1 ET, 2 PD, 1 other	Wrist and elbow	Co-contraction	9 ± 35%
Widjaja et al., 2011	1 ET	Wrist	Out-of-phase	57%
Gallego et al., 2013	4 ET, 2 PD	Wrist	Co-contraction	52 ± 25%
Bó et al., 2014	10 ET	Wrist and fingers	Co-contraction	60 ± 27%
Dosen et al., 2015	2 ET, 4 PD	Wrist	Out-of-phase	60 ± 14% (> thr_m_), 42 ± 5% (<thr_m_)
Jitkritsadakul et al., 2015	34 PD	Wrist	Co-contraction	44 ± 33%

*The table summarizes the previous studies investigating the use of electrical stimulation for tremor suppression with respect to patient group, target joint, stimulation strategy, and efficiency. In the two studies including “other” patients this referred to cerebellar tremor. In three of the studies (Popović Maneski et al., 2011; Bó et al., 2014; Dosen et al., 2015) one subject was excluded from the estimation of the average tremor suppression rate due to a lack of any effect of the stimulation. Thr_m_ denotes motor threshold*.

In a recent study (Dosen et al., 2015; Table 1), we observed significant levels of tremor suppression when stimulating muscles below the threshold of direct muscle activation via efferent fibers. This suggests that stimulation of afferent pathways may be an alternative tremor suppression strategy. The underlying neurophysiological mechanisms, however, were not identified and the influence of different stimulation settings on the suppression effect was not investigated. For these reasons, the primary aim of this study was to identify the most effective way to deliver the stimulation by testing different stimulation interfaces (surface and intramuscular) and by systematically varying the stimulation settings (pulse amplitude and timing). Furthermore, we hypothesized that differences in tremor suppression across stimulation settings could help to identify the physiological pathways through which tremor suppression occurred. For example, if the suppression is primarily achieved via cutaneous sensory afferents, which is a distinct possibility (Heo et al., 2015), then surface stimulation should produce superior results to intramuscular stimulation, and stimulation timing would be largely irrelevant. On the other hand, if activation of proprioceptive afferents (working via reciprocal inhibition pathways) is most important, then the appropriate timing of stimulation could be critical, and intramuscular stimulation would likely be the more comfortable, less distracting delivery method.

## Materials and methods

### Stimulation strategy

**Figure 1:**
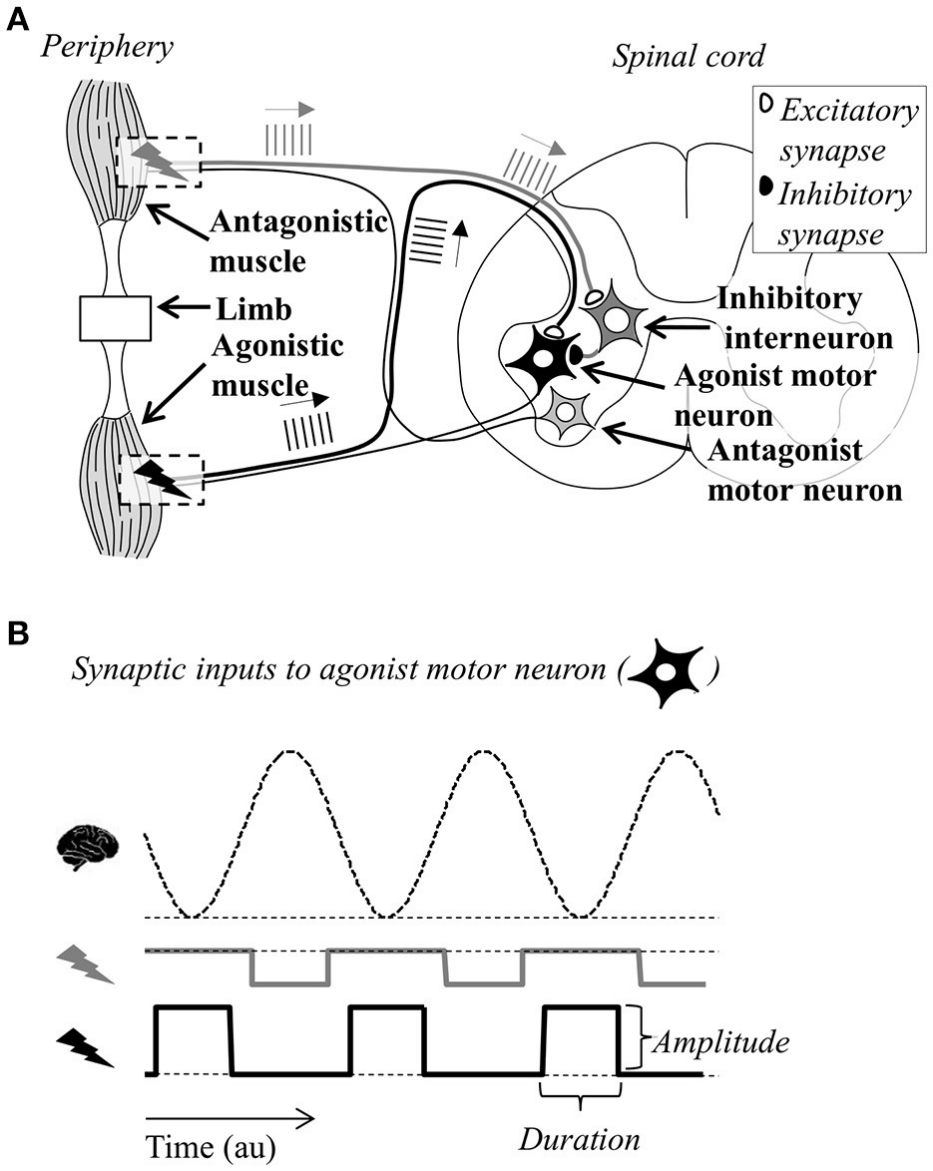
**Simplified, graphical representation of the tremor suppression strategy (not in scale)**. The antagonist muscles acting on the limb, and the relevant neural pathways (Ia monosynaptic, homonymous excitation, Ia polysynaptic, heteronymous inhibition, and motor neurons innervating the muscles) are depicted along with a cross-sectional view of the spinal cord **(A)**. Bursts of stimulation pulses above each of the two muscle bellies evoke trains of action potentials in the afferent fibers illustrated as vertical lines propagating toward the spinal cord. Afferent input to the motor neuron of the antagonistic muscle (light gray) is not included in the figure for clarity. By stimulating the two muscle bellies in opposite phases, the MN (black) receives inhibitory and excitatory input in an alternating manner **(B)**. In the figure, this input is represented in a simplified way as square pulses. The duration of the square pulse was represented as a percentage of the tremor period, while the amplitude was a function of stimulation frequency (determining the number of excitatory postsynaptic potentials from each synaptic bouton) and the stimulation intensity (the number of Ia fibers recruited). If this input is timed according to the dynamics of the descending oscillations causing the tremor (represented as a dashed sinusoid), the large fluctuations in the net input to the motor neuron and thus its output may be reduced, causing suppression of tremor.

First, we hypothesized that low-current, out-of-phase stimulation, as observed in Dosen et al. (2015), could result in tremor suppression through the neural mechanisms outlined in Figure 1. This potential explanatory model served as the starting point from the systematic investigation of the effect of different stimulation strategies on tremor suppression. Specifically, this conceptual model illustrates a mechanism by which stimulation of type Ia nerves innervating an antagonist muscle pair can lead to tremor suppression. The activation of this pathway results in excitation of the homonymous motor neurons (Schieppati, 1987) and inhibition of the motor neurons innervating the antagonist muscle (Wargon et al., 2006). In this way, the stimulation of this pathway can serve as a means to increase excitability in one motor neuron pool, while decreasing excitability in the motor neuron pool innervating its antagonist muscle. It is assumed that tremor can be described approximately as a reciprocal activation of the two motor neuron populations innervating the antagonist muscle pair (Raethjen et al., 2000; Milanov, 2001). Therefore, the properly timed afferent stimulation can be used to modulate the excitability of the motor neuron pools oppositely to that arising due to descending tremorogenic activation (supraspinal input). More specifically, if the Ia pathway of one muscle is activated during the tremorogenic EMG bursts in the other and vice versa, this stimulation would, in theory, serve to generate a more stable membrane potential for both motor neuron pools, as illustrated in Figure 1B.

This model requires selective stimulation of afferent fibers from individual muscles exhibiting tremor. Since this is not possible to achieve for all muscles with stimulation at superficial locations of mixed nerve trunks, the stimulation was delivered at the muscle belly or intramuscularly (see details in “Section Experimental Procedure”). A potential drawback of this method, however, is that stimulation at the muscle usually allows for activation of a smaller number of afferent fibers than nerve trunk stimulation (Bergquist et al., 2011, 2012).

It should be noted that this model is highly simplified since it does not account for several potentially relevant afferent pathways or the effects of the stimulation at supraspinal levels. However, we compared in the present study the effect of a stimulation strategy designed according to this model with other strategies (see Section Experimental Procedure). This allowed us to determine if the neuromodulation at the spinal level through activation of type Ia fibers (Figure 1) was likely to be the main mechanism underlying tremor suppression by delivering stimulation below the threshold of direct motor axon activation.

### Experimental procedure

Five PD patients (all male; 69.8 ± 7.0 years) and four ET patients (3 male, 1 female; 66.8 ± 5.0 years) exhibiting primarily wrist flexion/extension tremor participated in the experiment. In spite of differences in the pathophysiology of PD and ET, both patient groups were included for consistency with the previous tremor suppression studies (see Table 1). Tremor severity ranged from mild to severe [Fahn-Tolosa-Marin score (Fahn et al., 1993): 26.8 ± 5.9 (range 22–35) for ET patients and UPDRS score (Goetz, 2003): 17.8 ± 5.1 (range 9–21) for PD patients]. Patients were recruited by neurologists at the Hospital Universitario 12 de Octubre, Madrid, Spain, and provided written informed consent prior to participation. The ethical committee of the Hospital Universitario 12 de Octubre approved the experimental protocol.

The patients were randomly divided into two groups. For the first group (2 ET, 3 PD), electrical stimulation was delivered via surface electrodes (disposable ø3.2 cm; PALS Platinum, Axelgaard, US), whereas the second group (2 ET, 2 PD) received electrical stimulation through intramuscular electrodes. A multichannel stimulation unit (TremUNA, UNA Systems, SR) delivered current-controlled, biphasic charge-compensated pulses. The intramuscular electrodes were custom-made and consisted of a pair of Teflon-coated stainless steel wires (diameter 0.05 mm; A-M Systems, Carlsborg, WA) with uninsulated tips of 5 mm. The wires were inserted into the muscle via a 27-gauge hypodermic needle. For both groups, one surface electrode (disposable 5 × 7 cm; PALS Platinum, Axelgaard, US) positioned over the olecranon served as the common ground. For all patients, surface EMG was recorded in a bipolar configuration using standard ø11 mm Ag/AgCl electrodes (Neuroline 720, Ambu, DK) and an analog EMG amplifier (AnEMG12, OTBioelettronica, IT). The EMG signals were sampled at 1 kHz using a standard laptop equipped with a data acquisition card (NI-DAQ 6220, National Instruments, USA). Those signals were used to determine the presence of tremor and the centers of tremorogenic bursts and thus to time the stimulation timing. The laptop ran a tremor detection and suppression application developed in Microsoft Visual Studio for C# and Matlab 2012b (Mathworks, USA), as described in a previous study (Dosen et al., 2015). Quantification of tremor suppression was based on recordings of wrist movement, using an inertial measurement system (XBus kit, XSens, NL). The inertial data were sampled at 100 Hz. Two sensor units were secured to the dorsal side of the forearm and the hand with tape, aligned to the medial axis of the limb segments, and the flexion/extension wrist angle was computed as the difference between the recorded pitch angles of the segments. The data from the inertial units were used also to assess tremor characteristics at the baseline and compare this value between PD and ET patients.

The patient was seated comfortably in an adjustable chair, and the arm with the strongest wrist tremor was selected. Next, using a surface stimulation electrode, the optimal stimulation points for wrist flexor, and extensor muscles were identified as the locations at which pure flexion and extension, respectively, was achieved using 100 Hz stimulation at an intensity of a few mA and below the threshold for uncomfortable sensation. EMG electrodes were positioned on the muscle bellies identified by palpation during stimulation and a reference electrode (wristband) was placed around the wrist. For the patients receiving stimulation through intramuscular electrodes, the area was cleaned with alcohol prior to insertion, and the wires were afterwards secured using tape. The insertion point corresponded to the optimal location determined by probing using surface stimulation, as explained above. Stimulation on or within the muscle belly maximized muscle specificity and allowed a direct comparison to be made between surface and intramuscular stimulation.

H-reflex recruitment curves were obtained for the wrist extensor and flexor muscles in order to select the stimulation intensities eliciting a clear H-reflex. To determine the recruitment curve, eight pulses at each stimulation intensity (Dideriksen et al., 2015) were delivered (pulse width: 400 μs) with inter-pulse intervals of 4 s to minimize post-activation depression (Clair et al., 2011). First, 3, 6, 9, and 12 mA for surface stimulation and 1, 2, 3, and 4 mA for intramuscular stimulation, both in a randomized order (in most patients, additional stimuli was delivered later; see below). For some patients, the highest currents were omitted if they exceeded the threshold for discomfort. The EMG traces recorded after each of the eight stimuli at the same stimulation current were aligned according to the location of the stimulation artifact and averaged, and the H-reflex was estimated as the peak-to-peak amplitude at a latency of 20–35 ms after the stimulus (Baudry et al., 2010). Next, the H-reflex amplitude was normalized to the root-mean-square of the single EMG traces in the 50-ms interval following the H-reflex. This was done to compensate for across-trial changes in background motor neuron excitability, due to sudden onsets of brief periods with tremor or changes in the voluntary activation level related to slight changes in arm position. The normalized H-reflex recruitment curve was inspected and additional stimulation intensities were applied at selected currents to increase the resolution of the curve, if necessary. These current levels were selected so a clear representation of the ascending and descending parts of the H-recruitment curve was obtained. Due to the short distance between the stimulation and recording electrode, the amplitude of the M-wave could not be estimated due to a temporal overlap with the stimulation artifact. Therefore, the activation of motor axons cannot be ruled out, but the use of stimulation intensities on the ascending side of the H-reflex recruitment curve suggests that the primary effect of the stimulation was afferent rather than efferent.

After the H-reflex recruitment curves were identified for both muscles, the tremor suppression trials were initiated. For each patient, a suitable task was selected in which tremor would be present without generating excessive fatigue or discomfort. Typically, in ET patients, the hand was held outstretched against gravity with the forearm supported, whereas for PD patients the arm rested on a padded box on the table in front of the subjects with the hand hanging unsupported. In some cases, the patient was asked to perform a cognitive task (e.g., counting backwards) to provoke tremor. The system for tremor detection and suppression has been presented in detail previously (Dosen et al., 2015) and will only be briefly described here. Tremor was detected from the surface EMG and its phase was identified (Dideriksen et al., 2011). Trains of stimulation pulses were delivered timed to this phase, according to the strategy illustrated in Figure 1. Due to the contamination of EMG by stimulation artifacts, tremor demodulation could only take place when no stimulation occurred. Therefore, recording and stimulation were performed in a sequential manner (1-s recording window, 2-s stimulation window). Electrical stimulation was delivered only when tremor was detected in the preceding recording window.

The assessment of tremor suppression was performed in 150 s long trials, during which the system was turned off and on in 30-s windows. In this way, two periods with “System ON” were present in each trial. If tremor spontaneously ceased for prolonged periods during a trial, it was discarded and repeated. Stimulation parameters (stimulation intensity and burst duration) were varied systematically across 10 trials per patient. The patients were blinded to the stimulation condition. Stimulation trains at currents immediately below those required to produce an H-reflex for a single stimulus have been shown to elicit a response after a number of stimuli (Dean et al., 2014; Dideriksen et al., 2015). For this reason, stimulation intensity was set to the current evoking either the maximum H-reflex (termed high H-reflex) or the current at the onset of the ascending segment of the H-reflex recruitment curve (termed low H-reflex). Stimulation burst duration was set to 20 or 40% of the tremor cycle duration. Two trials with each of the four combinations of the settings were tested (total of eight trials). In these trials, stimulation frequency was 100 Hz and the center of the train of stimulation pulses was delivered 15 ms before the predicted center of the EMG burst to account for the conduction delay, as used for sub-threshold stimulation in our previous study (Dosen et al., 2015). In addition, two control trials were performed with a random timing of the stimuli at each of the two currents. In these trials, the pulses were delivered to both muscles continuously during the stimulation window but with a random inter-pulse interval ranging from 10 to 100 ms. In all trials, a pulse width of 400 μs was applied. If tremor was detected in only one muscle, the stimulation pattern of both muscles was timed according to this signal exclusively.

The 10 trials were divided into two blocks of five trials (one with random stimulation timing plus one of each of the four combinations of stimulation timing and intensity). Within each block the order of the trials was randomized. A break of at least 2 min was given between each trial. The division into two blocks was done to optimize the chances of completing at least one trial for each combination of the stimulation parameters, since we expected a substantial risk of the patient withdrawing from the experiment prematurely due to fatigue, or discarding the trial due to patient inability to tolerate the stimulation, or sudden cessation of tremor. The patients were blinded to stimulation condition.

### Data analysis

First, the relative delay between the EMG envelopes of the two muscles was estimated from the peak of their cross-correlation function (in periods without stimulation). In this way, it could be assessed to which degree the muscles exhibited out-of-phase tremor, which was an assumption underlying the proposed suppression strategy (Figure 1).

Next, the level of tremor suppression was estimated based on the joint angle as described by Equation (1).

(1) $$S=1-\frac{\int_{f\;=\;3}^{9}{\left|F\left(\alpha_{on}\right)\right|}^{2}}{\int_{f\;=\;3}^{9}{\left|F\left(\alpha_{off}\right)\right|}^{2}}$$

where *S* is the level of tremor suppression, F is the Fourier transform and α the joint angle in conditions where the stimulation is either on (α_*on*_) or off (α_*off*_). According to Equation (1) the tremor power was estimated as the integral of the power spectrum of the joint angle signal over the range of frequencies typical for pathological tremors (3–9 Hz; Deuschl et al., 1998). The level of tremor suppression was defined as 1 minus the ratio between the tremor power with and without stimulation (baseline). In this way, values of tremor suppression near 1 indicated almost perfect suppression whereas values near 0 implied no change in tremor power, and negative values indicated tremor enhancement with respect to the baseline. Using Equation (1), the average level of tremor suppression was computed over the whole trial. To determine the statistical significance of the obtained level of tremor suppression, each of the terms of Equation (1) (i.e., tremor power when the system was on ($\int_{f=3}^{9}{\left|F\left(\alpha_{on}\right)\right|}^{2}$) and off ($\int_{f=3}^{9}{\left|F\left(\alpha_{off}\right)\right|}^{2}$)) was estimated in 1-s, non-overlapping windows for each trial (60 windows with system on, 90 with system off). In this way, the tremor power when the system was on and off, respectively, was compared using a Kruskal-Wallis test. In addition, the difference in tremor suppression when considering only the 2-s stimulation windows as α_*on*_ was estimated to assess the impact of the sequential stimulation strategy.

The average levels of tremor suppression for each patient were calculated for all trials with the same settings, yielding six values per patient (one for each combination of stimulation intensity and timing, assuming that all trials with all combinations of settings were successfully completed in that patient). Next, the influence of different settings in each parameter was analyzed using Wilcoxon rank sum test. For example, this test compared all values (across all patients) obtained with low H-reflex with all values obtained with high H-reflex, and similarly for the other settings (stimulation modality, stimulation timing). Linear regression analysis was used to investigate the relation between tremor suppression level and H-reflex amplitude as well as tremor frequency, respectively. Furthermore, tremor characteristics including power at the baseline, the coefficient of variation and median frequency of the tremor power were compared across the two patient groups using the Student's *t*-test. Finally, the EMG recordings from the periods with system off were analyzed offline using the tremor detection algorithm to obtain the percentage of 1-s windows without detectable tremor. Paired *t*-test was used to compare the percentage of recording windows in which tremor was detected across periods with system on and off. For all tests, the level of significance was set to *p* < 0.05.

## Results

**Figure 2:**
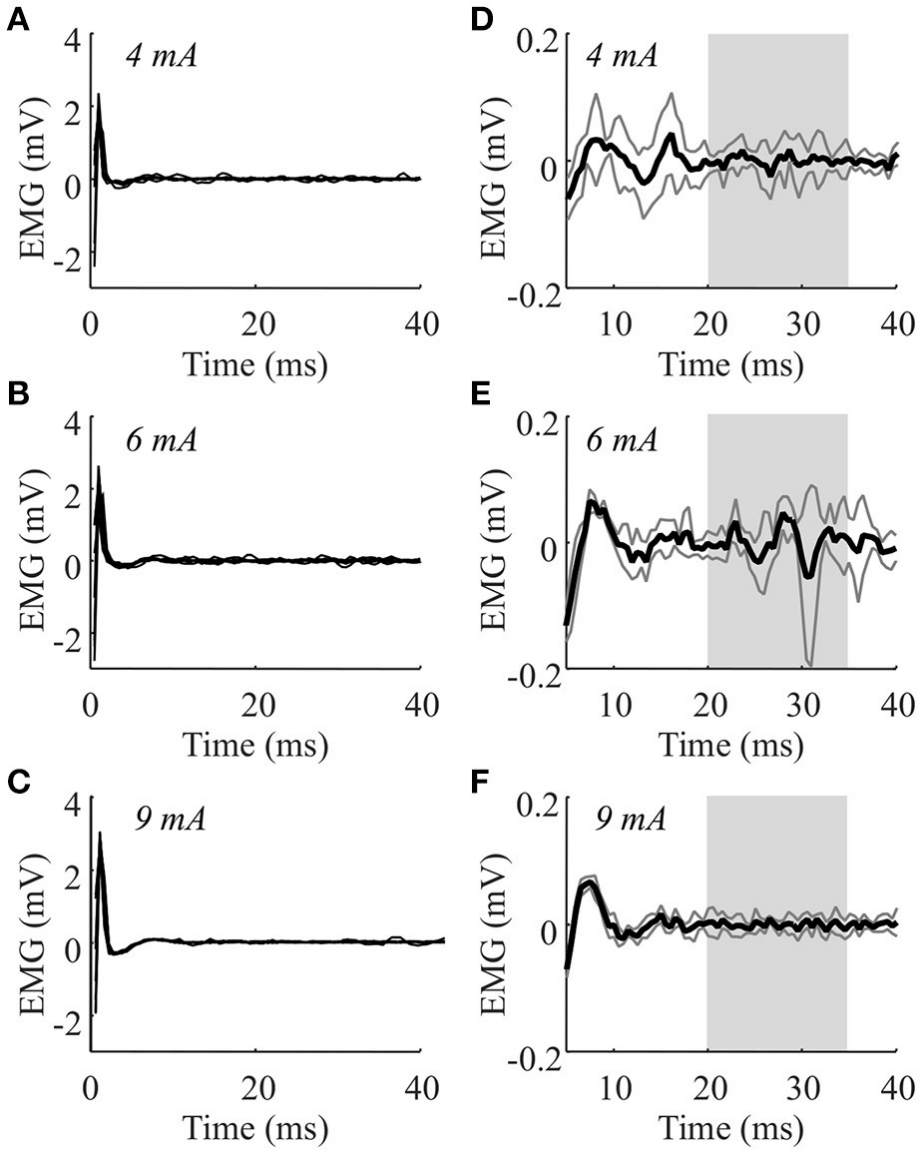
**The H-reflexes obtained from one patient (ET) using surface stimulation on the wrist extensor muscle**. The EMG during stimulation at 3, 4, 5, 6, 9, 12, and 15 rnA were recorded in this muscle of this patient. Panels **(A–C)** show the eight individual traces superimposed for 4, 6, and 9 rnA. Panels **(D–F)** show the averaged responses from the same stimulation intensities zoomed in to show the responses (dark gray lines indicate standard deviations). The H-reflex amplitude was identified as the peak-to-peak amplitude of the average response in the range 20–35 ms (light gray area).

Figure 2 shows a representative H-reflexes obtained using surface stimulation for one subject. Panels D-F show the averaged EMG traces in response to the eight stimuli at three selected stimulation intensities. The H-reflex amplitude (peak-to-peak amplitude in the time interval of 20–35 ms after the stimulus) were normalized to the baseline EMG level. For this muscle, 4 and 6 mA were selected (low and high H-reflex, respectively) for the suppression trials. At higher currents, the H-reflex decreased, as expected. The M-wave amplitude (delay <10 ms) could not be identified due to overlap with the stimulation artifact. Averaged across all patients, the maximum H-reflex amplitudes were 0.05 ± 0.01 mV for intramuscular stimulation and 0.06 ± 0.03 mV for surface stimulation. Maximum normalized H-reflex amplitudes were 2.34 ± 0.45 (intramuscular) and 2.06 ± 0.43 (surface). There was no systematic difference in the amplitudes across muscles. The current required for maximum H-reflex was 8.2 ± 2.5 mA (extensor) and 17.4 ± 6.1 mA (flexor) for surface stimulation and 1.1 ± 1.0 mA (extensor) and 0.5 ± 0.4 mA (flexor) for intramuscular stimulation.

Tremorogenic behavior in both flexor and extensor muscles was consistently detected in the EMG of five patients (2 ET, 3 PD), while it was detected for one of the muscles in the remaining patients. In these five patients, the delay between the successive tremorogenic bursts in each muscle expressed as a percentage of the tremor period was 32.8 ± 12.8% (range: 13.7–49.7%; 50% delay indicating perfect out-of-phase behavior). Tremor frequencies were 4.4 ± 1.0 Hz (PD) and 6.1 ± 0.9 Hz (ET).

**Figure 3:**
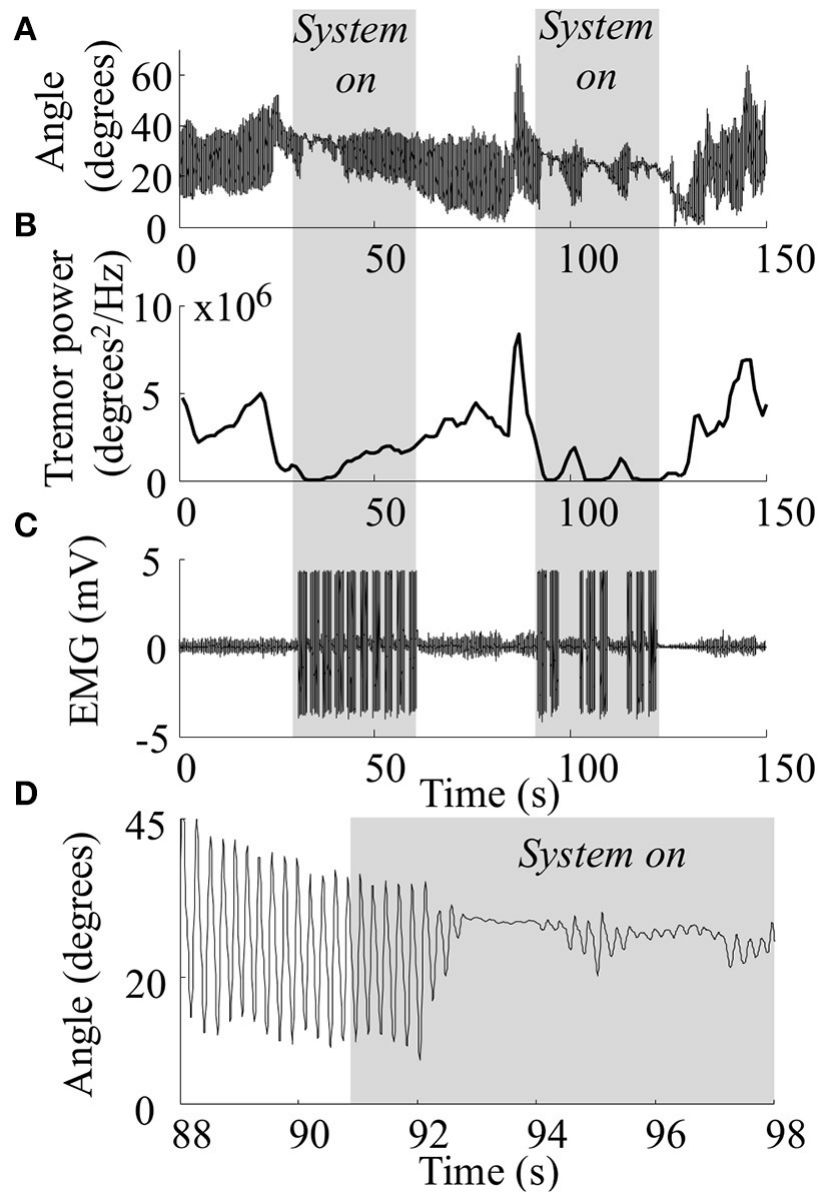
**Raw data collected during one tremor suppression trial for one PD patient with a high level of tremor suppression**. In this trial surface stimulation at a current evoking high H-reflex amplitude and a stimulation duration of 40% of the tremor period was used. Wrist angle (**A**: entire duration of trial; **D**: zoom on a 10 s period of the trial between “system off” and “system on”), wrist tremor amplitude **(B)**, wrist extensor EMG **(C)**. Gray areas indicate the periods in which the system was on, indicated clearly by large stimulation artifacts in the recorded EMG.

Figure 3 depicts an example of one tremor suppression trial (PD; surface stimulation; intensity: high H-reflex; burst duration: 40%). In the two 30-s time intervals in which the tremor suppression system was on, a clear decrease in tremor amplitude was observed. These intervals are visible in the recorded EMG as the periods contaminated by the stimulation artifacts (Figure 3C). The tremor amplitude varied within the two intervals with “system-on.” In the first of these intervals, the tremor was almost completely suppressed at first, but increased gradually over the next 30 s. This may be explained by an increase in the amplitude of the descending, tremorogenic synaptic input to the motor neurons during that interval. In the second interval, tremor was not detected in two recording windows (after 100 and 110 s), leading to increased tremor amplitudes at the onset of the next stimulation window. These events can be recognized from the recorded EMG (Figure 3C) as the two longer intervals without artifacts during the second system-on period, i.e., the system was on but it did not stimulate since tremor was not detected. It should be noted that in this patient, as well as across all patients, large variations in tremor amplitude were observed during the system-off periods, reflecting the dynamic nature of pathological tremor. Due to the sequential tremor suppression strategy, tremor amplitude increased slightly during the 1-s recording windows and decreased again once the stimulation started (Figure 3D).

Not all trials could be obtained from all patients, as explained before. In total, 76 trials (out of the 90 planned trials) were completed. 8.5 ± 1.9 and 8.4 ± 1.1 trials per patient were completed for intramuscular and surface stimulation, respectively, while 3.9 ± 1.3 and 4.6 ± 0.7 trials per patient were completed for the low and high H-reflex conditions, respectively. Across all completed trials, tremor was detected in 90.7 ± 8.2% of periods with system on and in 92.3 ± 7.9% of periods with system off (not significantly different; *p* = 0.75).

**Figure 4:**
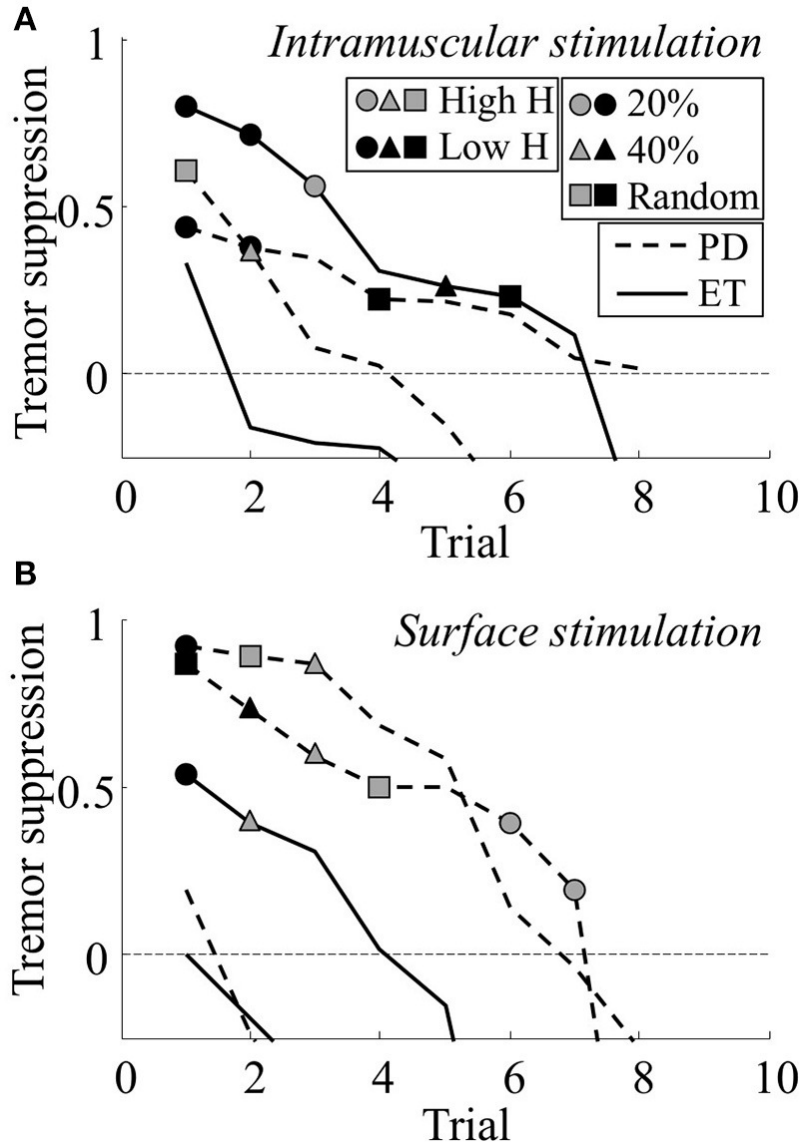
**The level of tremor suppression obtained across the different trials for all patients using intramuscular (A)** and surface **(B)** stimulation. The y-axis is truncated below –0.25. Several trials showed negative levels of tremor suppression below this point and are not shown in the plot. In both panels, each line indicates all trials from one patient (dashed line, Parkinson's Disease; unbroken line, Essential Tremor). The trials are ranked according to obtained suppression level (best trial first). Trials with statistical significant suppression are indicated with a symbol: Circles represent short stimulation duration (20% of tremor cycle), triangles represent long stimulation duration (40% of tremor cycle), and squares represents random stimulation timing. Symbol color indicates stimulation intensity: Gray represents stimulation at currents evoking high H-reflexes and black represents stimulation at currents evoking low H-reflexes.

Significant tremor suppression was observed in 21 trials (2.3 ± 2.2 per patient), distributed across 3/4 patients receiving intramuscular stimulation and 3/5 patients receiving surface stimulation. The tremor suppression levels for each trial are available as supplementary material. The trials with positive outcome (tremor suppression) are summarized in Figure 4. Here, the levels of tremor suppression for each patient are shown per trial, with the trials ranked according to the level of suppression achieved. In this way, the figure illustrates the tremor suppression level in the best trials as well as the cross-trial variability. The average of the highest suppression levels across all patients were 0.54 ± 0.20 (intramuscular) and 0.50 ± 0.41 (surface), respectively. There was no significant correlation between the highest level of tremor suppression and the the maximum amplitude of the H-reflex (*r*^2^ = 0.05) or the tremor frequency (*r*^2^ = –0.07). Also, whether tremor was present in one or both muscles did not predict the tremor suppression level [highest suppression: 0.48 ± 0.27 (both muscles) vs. 0.55 ± 0.37 (one muscle)]. When considering only the stimulation windows (thereby discarding the 1-s recording windows when the system was on), the tremor suppression level in the best trials improved for all but one patient, and was 0.58 ± 0.35 across all patients. Optimal tremor suppression was highest for PD patients [0.60 ± 0.30 (PD) vs. 0.41 ± 0.34 (ET)]. This may in part be explained by the fact that the baseline tremor power for PD patients was more stable [coefficient of variation: 113 ± 47% (PD) vs. 176 ± 90% (ET); significantly different: *p* = 0.002] and changed more slowly [median frequency: 0.09 ± 0.06 Hz (PD) vs. 0.16 ± 0.05 Hz (ET); significantly different: *p* < 0.0001], thereby allowing for more accurate predictions of tremor characteristics.

**Figure 5:**
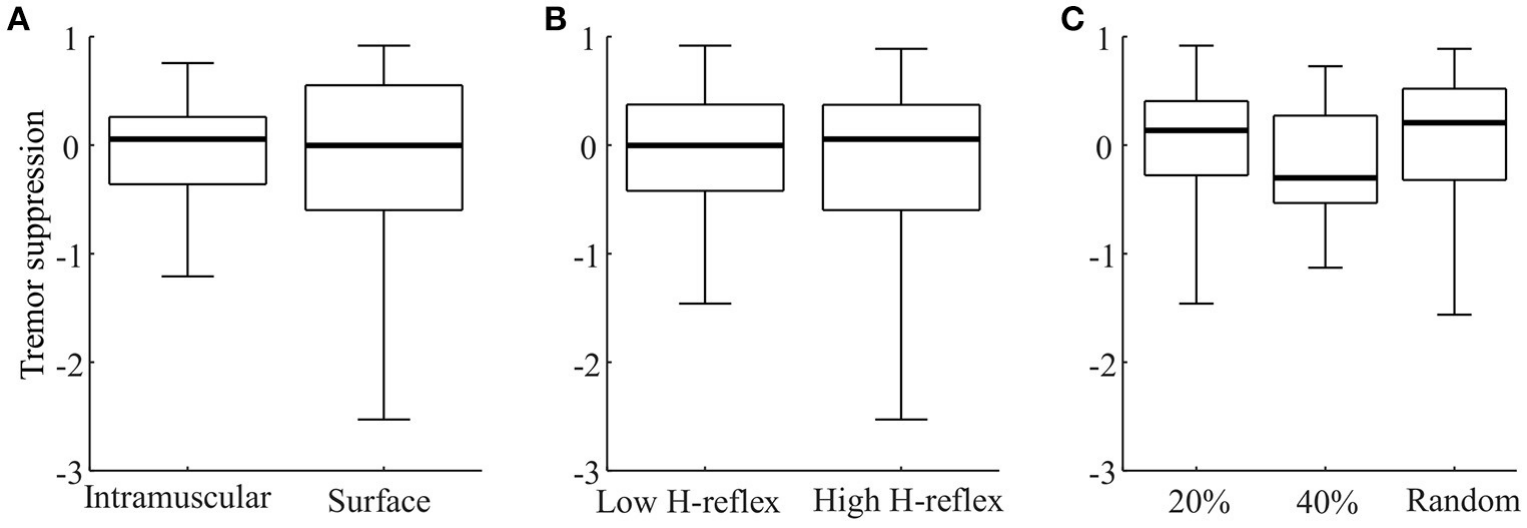
**Box-plot representations of the average suppression level for each stimulation setting** (**A**: stimulation modality; **B**: Stimulation intensity; **C**: Stimulation timing) across subjects. Each panel compiles 50 values. In each panel 3 values were excluded as outliers (was below 1.5 × inter-quartile range from the lower quartile).

Figure 5 shows the distributions of tremor suppression values across stimulation settings. Here, the average tremor suppression level from repeated trials was included as their average value. In four cases, no trials with one combination of settings were recorded. For this reason, Figure 5 compiles 50 values. Wilcoxon rank sum test indicated that there was no significant difference between the tremor suppression levels obtained across any of the different stimulation parameters (*p* > 0.4 in all cases). Table 2 represents a different way to compare the efficiency across settings, by summarizing the number of trials with significant tremor suppression for each combination of settings. Each setting produced significant tremor suppression in at least one trial, however, the majority of the settings also sometimes produced statistically significant tremor enhancement. From this point of view, the optimal stimulation protocol appeared to be intramuscular stimulation with short, low-intensity stimulation pulses (Table 2, gray fields). Here, 4/6 trials involved tremor suppression (two patients with significant suppression in both trials), whereas no difference in tremor power was observed in the remainder. Stimulation with random timing (applied with both intramuscular and surface stimulation), however, also enabled tremor suppression in several trials. An interesting observation was that surface stimulation always produced a greater number of trials showing tremor enhancement (35.0% of all trials), as compared with the same stimulation settings delivered via intramuscular electrodes (11.8% of all trials).

**Table 2:** **Tremor suppression across different settings**.

**Intensity**	**Low H-reflex**						**High H-reflex**					
**Duration**	**20% (*n*_i_ = 6, *n*_s_ = 6)**		**40% (*n*_i_ = 6, *n*_s_ = 9)**		**Random (*n*_i_ = 4, *n*_s_ = 4)**		**20% (*n*_i_ = 7, *n*_s_ = 9)**		**40% (*n*_i_ = 7, *n*_s_ = 9)**		**Random (*n*_i_ = 4, *n*_s_ = 5)**	
**Effect**	**Pos.(%)**	**Neg. (%)**	**Pos. (%)**	**Neg. (%)**	**Pos. (%)**	**Neg. (%)**	**Pos. (%)**	**Neg. (%)**	**Pos. (%)**	**Neg. (%)**	**Pos. (%)**	**Neg. (%)**
Intramuscular	67	0	17	17	50	0	14	14	14	14	25	25
Surface	17	50	11	33	50	25	22	44	33	44	40	20
Total	42	25	13	27	50	13	19	32	25	31	33	22

*The table shows the percentage of trials in which a statistically significant effect (reduction: positive or enhancement: negative; third row) of the tremor was obtained during the stimulation for each of the applied stimulation settings. Gray fields indicate conditions where the number of patients exhibiting significant reductions was higher than the number of patients in which the stimulation produced tremor enhancement. n indicates the number of trials, the subscripts i and s stand for intramuscular and surface stimulation*.

For the two stimulation modalities, successful repetitions of trials with the same stimulation parameters were carried out in 12 cases for each modality (i.e., 24 pairs of trials out of the 32 pairs of trials that could maximally be obtained). The variability in performance across the repetitions was lower with intramuscular stimulation [median inter-trial variability: 0.30 (intramuscular) vs. 0.87 (surface)], suggesting that this type of stimulation produces a more consistent effect on tremor.

## Discussion

In this study, we investigated suppression of pathological tremor through stimulation of afferent fibers. This was tested using several combinations of stimulation parameter settings in 9 patients with ET or PD in order to systematically explore the parameter space and identify the most effective combinations. Stimulation intensities were determined based on H-reflex recruitment curves suggesting that the afferent volleys induced by the stimulation were at least partly conveyed via type Ia fibers. In this way, the study represents a first step toward understanding if afferent stimulation can effectively suppress tremor, and if this strategy can be optimized. To the best of our knowledge, this is the first study investigating tremor suppression using sensory stimulation that proposes a systematic method for selection and assessment of the stimulation parameters. Overall, statistically significant tremor suppression was achieved in the majority (6/9) of the patients, with an average magnitude of 52% in the optimal cases for each patient (Figure 4). This level is higher than we previously observed using a more common approach (Dosen et al., 2015), indicating a potential utility for patient-specific stimulation protocols. The variability in suppression level across subjects, however, was high, with some patients responding very well to the stimulation (e.g., three patients had tremor suppression levels above 75%), whereas other patients hardly responded at all. The suppression levels of the best responders is thus comparable to the highest previously reported for efferent stimulation (Gillard et al., 1999) (83% in 3 PD patients). In this context, it is important to note that motor stimulation, unlike afferent stimulation, has several undesired side-effects, such as excessive muscle fatigue and discomfort/pain (Maffiuletti, 2010; Bickel et al., 2011). We did not, however, find any consistent relation between stimulation parameter setting and suppression level across the subjects (Figure 5). The stimulation parameter settings resulting in optimal suppression differed among subjects, and almost any one combination of settings could yield both positive and negative results across patients (Table 2). Although there was no consistent relation between parameters and suppression levels, the data suggests that intramuscular stimulation at low intensities (current evoking low H-reflex amplitude) and short durations (20% of tremor period) might be the most promising combination. Since the M-wave could not be discriminated, it cannot be ruled out that some level of motor axon stimulation was present. However, the fact that the lowest intensity tended to be the most efficient suggests that substantial levels of efferent stimulation were not required in order to achieve tremor suppression as stimulation at low currents is less likely to activate efferent fibers.

In this study, tremor suppression was compared when using surface and intramuscular stimulation. Here, the underlying assumption was that intramuscular stimulation involved a lower level of stimulation of cutaneous afferents, since it was delivered deep within the muscle and further away from the skin. Therefore, this would be the best method to achieve more selective stimulation of type Ia fibers according to the proposed conceptual scheme (Figure 1). The results indicated that the out-of-phase stimulation strategy could be efficient for both types of stimulation. Intramuscular stimulation, however, provided a more repeatable outcome and was less likely to increase the tremor amplitude during the stimulation. This could be related to lower activation of cutaneous afferents. For example, surface stimulation with the high current level may have been perceived as more intense, as the current passes through the skin activating tactile sensors. This may have led to changes in anxiety or focus, which are subjective factors known to have an effect on tremor magnitude (Deuschl et al., 1998). Another explanation, relevant in particular to the repeatability, is that activation of the same group of nerve fibers across trials is more likely with intramuscular stimulation, since the electrode-nerve interface is less affected by the movement of the skin with respect to the underlying muscle. Conversely, in surface stimulation, the electrode moves with the skin to which it is attached and the stimulation effect varies over time. This is an important outcome suggesting that, in the future, a fully implantable solution exploiting our proposed neuromodulatory approach might be an effective solution for tremor suppression. Interestingly, some patients exhibited high levels of tremor suppression when exposed to randomly timed stimulation. This implies that mechanisms that do not depend on stimulation timed to the tremor phase (as the envisioned strategy for neuromodulation; Figure 1) can contribute to tremor alleviation. It is well-established that thalamic deep brain stimulation (Kalia et al., 2013) as well as stimulation of the dorsal column of the spinal cord (Fuentes et al., 2009) can desynchronize the neural activity causing several PD symptoms including tremor. Continuous surface stimulation of muscles at levels below motor threshold is also effective for tremor suppression, and may even last for several minutes after stimulation has been stopped (Heo et al., 2015). It can be hypothesized that our randomly timed stimulation may have evoked similar tremor suppression pathways as in Heo et al. (2015), although it is likely that such pathways are better stimulated with continuous rather than random stimulation. The effectiveness of appropriately-timed stimulation in our study demonstrates that Ia inhibitory pathways can be recruited for the purpose of tremor suppression. Conceptually, the strategy outlined in Figure 1 would still work even if only one muscle of an antagonist pair receives pathological oscillatory drive, which was the case in some of our patients. Accordingly, our results demonstrated that tremor suppression could be achieved using out-of-phase stimulation even where tremor was not present both muscles.

Although our randomly-timed stimulation condition can be viewed as a kind of sham, to control for the effects of stimulation in general, the fact that sensory stimulation may influence tremor (e.g., Heo et al., 2015) underscores the need for a new kind of sham/placebo condition in future mechanistic studies. Because sensory stimulation is perceivable by patients, it is possible that unintended effects of anxiety, surprise, distraction, etc. could have temporary effects on tremor which are more psychological in nature rather than attributable to particular afferent pathways. Perhaps stimulation of the leg or the opposite limb could provide this type of control. If a psychological mechanism were at work, one might expect the highest amplitudes of stimulation to consistently suppress tremor more than low amplitudes, and out-of-phase timing should have essentially no advantage over random timing. Since this is not what we observed, we favor the interpretation that stimulation does have its suppressive effects via afferent pathways.

There are several ways in which the proposed tremor suppression strategy may be developed further to potentially increase its performance. When considering only the time intervals during which stimulation was delivered (excluding recording windows; see Section Experimental procedure), the average optimal tremor suppression improved (58%). This suggests that the performance may be further increased by minimizing the recording period relative to the stimulation period, or by allowing continuous stimulation by removing the stimulation artifacts in the EMG through hardware blanking or filtering (Hartmann et al., 2015). Alternatively, the tremor detection could rely on mechanical signals as in other studies (Popović Maneski et al., 2011; Gallego et al., 2013), which would imply that the ability to detect tremor would be unaffected by the stimulation. A drawback of this approach, at least for our proposed tremor suppression scheme (Figure 1), is that careful timing of stimulation requires identification of tremor from individual muscles rather than limb movements. Importantly, our method is radically different with respect to other approaches to suppress tremor using efferent but also afferent stimulation. We deliver the afferent input to produce a timed neuromodulation at the spinal level, and the input needs to be synchronized with respect to the descending oscillatory command, as explained in Figure 1. This information (tremorogenic command) cannot be obtained using a mechanical sensor, but only by demodulating the EMG, as implemented in the present system. Stimulation with accurate timing is particularly important in patients where the tremor is not limited to a single degree-of-freedom and when the tremorogenic activation patterns across muscles are not perfectly in or out of phase. In addition, EMG-based analysis enables detection of voluntary muscle activity (Dideriksen et al., 2011), which would allow tremor suppression systems to stimulate only during functional tasks. This could potentially minimize patient discomfort and prolong battery life. The fact that the optimal stimulation parameters varied widely across subjects suggests that an improvement in the tremor suppression may be achieved by finding the optimal, patient-specific setting. Due to the large number of combinations of stimulation parameters (intensity and burst duration), it was not possible to exhaustively test and compare all relevant combinations in this study. Instead, if implemented in a wearable neuroprosthetic device, it is possible that a more systematic investigation of this solution space can be carried out over long periods of use.

The large difference in tremor suppression level across patients could suggest that only a subset of all tremor patients can benefit from this type of stimulation. Further, its efficiency may be impaired by neural adaptations over prolonged use. For example, in PD patients, a severity-dependent depression of the H-reflex has been shown (Hiraoka et al., 2005), and H-reflexes in general have been reported to be depressed in the aging population (Scaglioni et al., 2002). Accordingly, the observed H-reflex amplitudes were relatively small with respect to the baseline EMG activity and could only be detected by averaging multiple responses. However, the fact that tremor suppression was often observed at stimulation intensities below those required for a high H-reflex amplitude (Table 2) suggests that the suppression mechanism was not simply mechanical activation of the muscle via an indirect (afferent) pathway. That said, the efficacy of Ia activation may be influenced by frequency of stimulation as well as intensity. For example, it is possible that the post synaptic potentials onto motor neurons or interneurons could be reduced by post-activation depression or presynaptic inhibition. However, in healthy, young subjects, the H-reflex has been shown to be relatively stable for 20 (Clair et al., 2011) and 100 Hz (Dideriksen et al., 2015) in up to 20 consecutive stimuli.

In summary, this study investigated suppression of pathological tremor using electrical stimulation of afferent pathways. While significant tremor suppression was observed in 6/9 subjects, and levels of suppression >75% were achieved in a subset of patients, it was evident that our approach requires personalized stimulation settings in order to obtain optimal results. Further research in a larger patient population with long-term recordings of daily-life activities is needed to verify the applicability of the approach in portable, neuroprosthetic devices.

## Ethics statement

This study was carried out in accordance with the recommendations of Hospital Universitario 12 de Octubre, Madrid, Spain with written informed consent from all subjects. All subjects gave written informed consent in accordance with the Declaration of Helsinki. The protocol was approved by the the ethical committee of Hospital Universitario 12 de Octubre, Madrid, Spain.

## Author contributions

JD, CL, SD, and SM contributed to data acquisition and analysis. JD, CL, SD, SM, ER, JP, JB, and DF contributed to the conception of the work, interpretation of data, and drafting and revising the manuscript. Furthermore, JD, CL, SD, SM, ER, JP, JB, and DF approved the manuscript for submission and agreed to be accountable for all aspects of the work in ensuring that questions related to the accuracy or integrity of any part of the work are appropriately investigated and resolved.

## Funding

This work has been supported by the Commission of the European Union through the grant ICT-2011-287739 (NeuroTREMOR).

### Conflict of interest statement

The authors declare that the research was conducted in the absence of any commercial or financial relationships that could be construed as a potential conflict of interest.
